# Tensile Strength Prediction of Short Fiber Reinforced Composites

**DOI:** 10.3390/ma14112708

**Published:** 2021-05-21

**Authors:** Zheng-Ming Huang, Wei-Jing Guo, Hong-Bo Huang, Chun-Chun Zhang

**Affiliations:** School of Aerospace Engineering & Applied Mechanics, Tongji University, 1239 Siping Road, Shanghai 200092, China; 1830899@tongji.edu.cn (W.-J.G.); 2110153@tongji.edu.cn (H.-B.H.); 1631800@tongji.edu.cn (C.-C.Z.)

**Keywords:** short fiber composite, micromechanics, stress concentration factor, true stress, tensile failure

## Abstract

Essentially, every failure of a short fiber reinforced composite (SFRC) under tension is induced from a matrix failure, the prediction of which is of fundamental importance. This can be achieved only when the homogenized stresses of the matrix are converted into true values in terms of stress concentration factors (SCFs) of the matrix in an SFRC. Such an SCF cannot be determined in the classical way. In this paper, a closed-form formula for the longitudinal tensile SCF in the SFRC is derived from the matrix stresses determined through an elastic approach. The other directional SCFs in an SFRC are the same as those in a continuous fiber composite already available. A bridging model was used to calculate the homogenized stresses explicitly, and a failure prediction of the SFRC with arbitrary fiber aspect ratio and fiber content was made using only the original constituent strength data. Results showed that the volume fraction, the aspect ratio, and the orientation of the fiber all have significant effect on the tensile strength of an SFRC. In a certain range, the tensile strength of an SFRC increases with the increase in fiber aspect ratio and fiber volume content, and the strength of the oriented short fiber is higher than that of the random short fiber arrangement. Good correlations between the predicted and the available measured strengths for a number of SFRCs show the capability of the present method.

## 1. Introduction

Short fiber (standing for the reinforcement) and particle reinforced composites have been widely used in industries, due to their excellent machinability and good mechanical performance [1,2,3]. Tensile strength is one of the most important mechanical properties for applications. Accurate prediction for the tensile strength of an SFRC plays an important role in the design of a structure made with such composites. In addition, when the fiber aspect ratio (length over diameter) changes, an SFRC can be degraded to a continuous fiber composite or a particle reinforced composite, therefore the study of SFRCs is of universal significance. However, due to the discontinuous feature of the reinforcement, the stress distribution in an SFRC is more complicated than that in a continuous fiber composite, and a variation in the fiber content, aspect ratio, and orientation makes the strength prediction even more difficult [4]. Existing models established so far to predict the tensile strength of an SFRC can be broadly classified into four categories, i.e., modified rule of mixture (ROM) methods [5,6,7,8,9,10,11,12], laminate analogy approaches [13,14,15,16,17,18], shear-lag models [13,19,20,21,22], and finite element methods (FEMs) [23,24,25,26,27].

One of the most widely used approaches to tensile strength was developed from modifications on the rule of mixture. A coefficient of less than one is applied in front of the fiber strength to represent a reduction in all the influencing factors on the fiber load sustaining ability. When modifying the ROM, most scholars focus on the effect of fiber length and orientation. Fu et al. [5,6,7] established a fiber orientation distribution function to consider the random distribution of fiber’s orientations. Taha et al. [8,9,10] directly introduced the correction factors of fiber length efficiency to reflect the effect of fiber discontinuity. However, prediction accuracy of the tensile strength is not as desirable as that of the modulus. Kelly-Tyson’s theory [11], a commonly used model, was found to overestimate the strength of an SFRC [12]. In addition, the model usually needs the correlation coefficients for fiber length and orientation distributions, which are generally obtained through fitting the experiments.

A “Laminate Analogy” approach postulates that a composite reinforced with short fibers distributed randomly in-plane is made of a series of thin UA (uniaxially aligned) fiber plies bonded together with different fiber orientation angles [13]. To consider the effect of fiber orientations, Chen [14] and Hahn [15] used this method and tried to incorporate three failure modes into a solution. The strength of the SFRC is obtained by averaging the off-axis composite strength over a range of *π*/2. Shokrieh et al. [16] combined the laminate analogy (LA) approach with a progressive damage model to study the fiber aspect ratio on the tensile strength of GNP/nanocomposites. Results showed that when the fiber aspect ratio was less than 500, the strength of the nanocomposites increased obviously with the increase in GNP aspect ratio. However, due to the difficulty in preparing UA SFRC, the strength of a thin layer in its local coordinate system was not directly available. Most researchers used the modified rule of mixture to determine the axial strength. The transverse strength was taken as that of the matrix, and the shear strength was specified as 1/2 or $1/\sqrt{3}$ of the tensile strength of the matrix [17,18].

The shear-lag model was proposed by Cox [19], on the basis of load transfer theory, which showed that the stress applied to the composite is transferred to the fibers through interfacial shear stress [20]. Desirable predictions for the tensile strength of an SFRC with a well-bonded interface can be found [21]. Based on this model, Zhang [22] obtained an interfacial debonding stress and combined it with the ROM to predict the axial tensile strength of an SFRC. To account for the effect of fiber orientation, Shokrieh et al. [13] combined the shear-lag model with the laminate analogy method to predict the failure and strength of two-dimensional, randomly oriented SFRCs. Additionally, the shear-lag theory was used to determine the longitudinal strength of a single-layer SFRC. However, this model only calculates a longitudinal stress transfer, and can only predict the axial tensile strength.

In recent years, finite element simulation has become more and more important, because it can easily simulate the mechanical behavior of an SFRC with arbitrary fiber length, fiber content, and fiber orientation. In addition, the FEM (finite element method) can eliminate some basic assumptions of the theoretical models, for example, a perfect interface bonding assumption [23]. Taking the fiber distribution into account, Hom et al. [24,25] constructed two finite-element models with the fiber ends aligned in one model and staggered in another to determine the fiber arrangement that best reflected the mechanical properties of an SFRC. Results showed that when fiber staggered each other, the predicted stress–strain response of the composite agreed well with the experimental results. To study the effect of fiber length on the tensile strength of an SFRC, a planar geometric model was used by Hashimoto [26]. Results showed that the tensile strength increased from fast to slow with the increase in fiber length. Although detailed stress distributions can be obtained through an FEM, it is still difficult to analyze the tensile strength of SFRCs due to the difficulty in determining the strength parameters of the constituent materials [27].

In a continuous fiber reinforced composite (CFRC), if the fiber strength is higher than that of the matrix, a fiber failure may possibly occur only when a dominant longitudinal load is applied. However, due to the high stress concentration of the matrix at fiber ends, the failure of a UA SFRC under longitudinal tension must result from a matrix failure. Furthermore, an interface debonding between the fiber and matrix is due to a matrix failure as well [28]. Thus, given that the fibers are stronger than the matrix, every tensile failure of an SFRC is essentially caused by a matrix failure. Any difficulty in predicting an SFRC’s tensile failure is almost always attributed to the inaccurate prediction of a matrix failure.

Recently, we have found that the micromechanics bridging model originally developed for CFRCs can be also applied to SFRCs [29]. Even though the homogenized internal stresses in the matrix and fiber of a composite can be explicitly available by bridging models, they must be converted into true values before a failure assessment can be carried out [30,31]. The stresses of fiber are uniform; therefore, the fiber’s true and homogenized stresses are the same [32,33]. The matrix’s true stresses are obtained by multiplying its homogenized counterparts with the SCFs. We have already obtained all of the SCFs of the matrix in a CFRC [28,30,31,34], and failures can be estimated from the concept of true stress [34]. Thus, for the failure and strength analysis of an SFRC, the critical step is in determining the matrix SCFs. In fact, only the axial SCFs of the matrix in an SFRC need to be determined, because the matrix’s SCFs in other directions are essentially the same as those of a CFRC. Very recently, we have preliminarily tried to obtain the longitudinal matrix SCF in an SFRC based on the FEM [35]. In [35], a 2D axial symmetry geometry representing the representative volume element (RVE) of an SFRC was established. A uniform displacement was imposed at the matrix end, and the resulting stress field of the matrix was used to determine the longitudinal tensile SCF of an SFRC. However, a numerical evaluation for the longitudinal SCF in any SFRC is inconvenient for application, because all of the other directional SCFs can be calculated by analytical formulae.

In this study, the stress field of the matrix was precisely obtained through an elastic approach. The exact stress field was then used to define the longitudinal tensile SCF of the matrix in an SFRC. Different from the definition of SCF in a CFRC, the RVE of the SFRC must be divided into three parts (i.e., a central segment and two end ones) according to the fiber aspect ratio, so that the SCF of the matrix tends to unity when the SFRC becomes a CFRC. The correctness of the longitudinal tensile SCF formula is double-checked against the numerical approach [35]. Based on the bridging model and the obtained longitudinal as well as other directional SCFs, the matrix’s true stresses are obtained. Whereas the tensile failure a UA SFRC can be easily assessed in terms of the true stresses, the strength of an SFRC with random fiber orientation can be predicted by subdividing the random SFRC into a series of UA SFRCs. Good correlation between the predicted results of this model and the existing experimental data for a number of SFRCs indicates that this model is valid for the strength prediction of such composites.

## 2. Homogenized Internal Stresses

In any mechanics of continuum media, the stress of any point in a material is defined as an averaged value of an infinitesimal element containing the point through [34]

(1) $$\sigma_{i}=\frac{1}{V\prime }(\int_{V\prime }{\tilde{\sigma }}_{i}dV)$$

**Figure 1:**
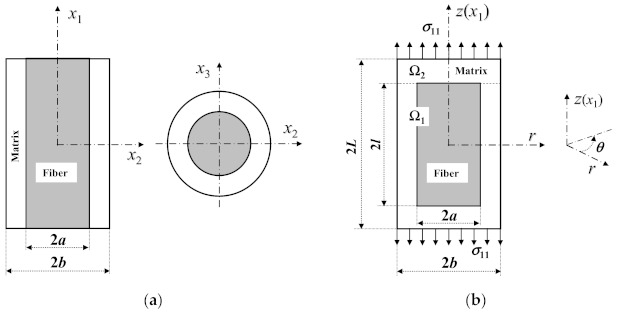
(**a**) RVE of a CFRC; (**b**) RVE of an SFRC.

The resulting stress, ${\tilde{\sigma }}_{i}$, is named as a point-wise value. *V*′ is the volume of the representative volume element (RVE). For a composite, however, one cannot take an infinitesimal element, because both the fiber and matrix must be simultaneously contained. Thus, a composite stress is defined, by nature, as a homogenized one through Equation (2), as long as only two constituents are involved [34].

(2) $$\sigma_{i}=\frac{1}{V\prime }(\int_{V\prime }{\tilde{\sigma }}_{i}dV)\equiv V_{f}\sigma_{i}^{f}+V_{m}\sigma_{i}^{m}$$

where *f* and *m* represent the fiber and matrix, and *V* is a volume fraction. $\sigma_{i}$ represents the homogenized stress. Based on the fundamental definition, the bigger the used RVE, the more the departure of the homogenized stress from the real stress. One of the smallest RVEs for a CFRC is indicated in Figure 1a, whereas that for an SFRC is shown in Figure 1b. The cell domain of an SFRC, represented by Ω, is divided into two parts: a fiber region Ω_1_(0 ≤ *z ≤ l* in the upper part), and a fiber end region Ω_2_(*l* < *z ≤ L* in the upper part).

Using a bridging equation [34],

(3) $$\left\{\sigma_{i}^{m}\}=[a_{ij}]\{\sigma_{j}^{f}\right\}$$

where [*a_ij_*] is a bridging tensor to be defined later, the homogenized internal stresses in the fiber and matrix are given by [36]

(4) $$\left\{\sigma_{i}^{f}\right\}={\left(V_{f}[I]+V_{m}[a_{ij}]\right)}^{-1}\left\{\sigma_{j}\right\}$$

(5) $$\left\{\sigma_{i}^{m}\right\}=[a_{ij}]{\left(V_{f}[I]+V_{m}[a_{ij}]\right)}^{-1}\left\{\sigma_{j}\right\}$$

[*I*] is a unit tensor. $\left\{\sigma_{j}\right\}$ is the external load applied on a composite.

Comparing Figure 1b with Figure 1a, it can be seen that the RVE of a short fiber composite is different from that of a continuous one only along the longitudinal direction. Thus, by bridging model, the non-zero bridging tensor elements of [*a_ij_*] are expressed as [29,36]

(6) $$a_{11}=\left\{\begin{array}{c} \frac{E^{m}}{E_{11}^{f}},for\;a\;continuous\;fiber\;composite\\ \frac{V_{f}E^{m}}{V_{m}E_{11}^{f}}\frac{\sigma_{11}L-E_{11}^{f}\left[\varepsilon_{L}^{1}l+\varepsilon_{L}^{2}(L-l)\right]}{E^{m}\left[\varepsilon_{L}^{1}l+\varepsilon_{L}^{2}(L-l)\right]-\sigma_{11}L},for\;a\;short\;fiber\;composite \end{array}\right.$$

(7) $$a_{12}=a_{13}=\frac{S_{12}^{f}-S_{12}^{m}}{S_{11}^{f}-S_{11}^{m}}(a_{11}-a_{22})=\frac{E_{11}^{f}\nu^{m}-E^{m}\nu_{12}^{f}}{E_{11}^{f}-E^{m}}(a_{22}-a_{11})$$

(8) $$a_{22}=a_{33}=a_{44}=\beta +\left(1-\beta \right)E^{m}/E_{22}^{f}$$

(9) $$a_{55}=a_{66}=\alpha +\left(1-\alpha \right)G^{m}/G_{12}^{f}$$

In the above equations, $E_{11}^{f}$, $E_{22}^{f}$ and $G_{12}^{f}$ are longitudinal, transverse and in-plane shear moduli of the fiber, respectively, and $\nu_{12}^{f}$ is the fiber’s longitudinal Poisson’s ratio. *E^m^*, *G^m^* and *ν^m^* are the Young’s and shear moduli, and Poisson’s ratio of the matrix, respectively. *σ*_11_ is an axial stress applied on the short fiber composite (Figure 1b). $S_{ij}^{k}$ refers to the compliance tensor element of the fiber (*k* = *f*) or matrix (*k* = *m*). $\varepsilon_{L}^{1}$ and $\varepsilon_{L}^{2}$ are the longitudinal homogenized strains of the domains Ω_1_ and Ω_2_ in Figure 1b, solutions to which are given in Appendix A. *l* and *L* represent half of the length of the fiber and matrix, respectively. It is noted that when *l* tends to *L*, the expression in Equation (6) for a short fiber composite becomes that for a continuous one [29]. Furthermore, the bridging parameters in Equations (8) and (9) assume *β* = *α* = 0.3 [34] for a CFRC, while taking *β* = *α* = 0.5 [29] for a short fiber or particle composite. All of the other bridging tensor elements not listed are zero.

## 3. True Internal Stresses

The homogenized internal stresses of the fiber and matrix obtained from the bridging model, or any other micromechanics model, in fact, cannot be directly used for failure detections. Instead, they must be converted into true values. Otherwise, the predicted strength will be much greater than the measured counterpart. Considering, for example, an E-Glass/LY556 UD (unidirectional) composite subjected to transverse tension *σ*_22_. The resulting composite failure is generally caused by a matrix tensile failure. According to the fiber and matrix properties provided in [37], and as shown in Table 1, the non-zero transverse stress of matrix obtained from the bridging model is given by $\sigma_{22}^{m}$ = 0.442*σ*_22_. Then, the transverse tensile strength is $\sigma_{22}^{u,t}$ = $\sigma_{u,t}^{m}$/0.442, with $\sigma_{u,t}^{m}$ = 80 MPa being the tensile strength of the LY556 matrix [37]. One then has $\sigma_{22}^{u,t}$ = 181 MPa, 5.2 times greater than the measured value, 35 MPa [37]. It is the stress concentration in the matrix after adding the fiber that gives this result.

The fiber stress field is uniform [33], therefore there is no change between its homogenized and true stresses. For the matrix, the conversion is achieved by multiplying its homogenized stresses with its SCFs. However, such an SCF cannot be defined following a classical means any more. Otherwise, the resulting SCF would be infinite when the interface between the fiber and the matrix cracks. An SCF of the matrix in a composite cannot be defined from a point-wise stress; therefore, it must be defined based on an averaged value. The classical definition for an SCF is a point-wise stress divided by an overall applied value, which is essentially a 2D value. By similarity, an SCF of the matrix must be defined as a line-averaged stress (1D quantity) divided by a volume-averaged stress (3D quantity), because the maximum available geometric dimensions are three. For the RVE of a composite, the integral line is along the outward normal direction of the failure surface of a composite, starting from the fiber end and ending at the end of the matrix [31].

When the fiber and matrix are in perfect interface bonding, the transverse tensile, transverse compressive, longitudinal shear, and transverse shear SCFs of the matrix, $K_{22}^{t}$, $K_{22}^{c}$, *K*_12_ and *K*_23_, respectively, are calculated through the following formulae [28,30,31].

(10) $$K_{22}^{t}=K_{33}^{t}=\left[1+\frac{\sqrt{V_{f}}}{2}A+\frac{\sqrt{V_{f}}}{2}(3-V_{f}-\sqrt{V_{f}})B\right]\frac{(V_{f}+0.3V_{m})E_{22}^{f}+0.7V_{m}E^{m}}{0.3E_{22}^{f}+0.7E^{m}}$$

(11) $$K_{22}^{c}=\left\{1-\frac{\sqrt{V_{f}}}{2}A\frac{\sigma_{u,c}^{m}-\sigma_{u,t}^{m}}{2\sigma_{u,c}^{m}}+\frac{B}{2(1-\sqrt{V_{f})}}\left[-V_{f}^{2}\left(1-2{\left(\frac{\sigma_{u,c}^{m}-\sigma_{u,t}^{m}}{2\sigma_{u,c}^{m}}\right)}^{2}\right)+\right.\right.\frac{\left(\sigma_{u,c}^{m}+\sigma_{u,t}^{m}\right)V_{f}}{\sigma_{u,c}^{m}}\left(1+\frac{\sigma_{u,c}^{m}-\sigma_{u,t}^{m}}{\sigma_{u,c}^{m}}\right)\;\left.\left.-\sqrt{V_{f}}\left(\frac{\sigma_{u,c}^{m}-\sigma_{u,t}^{m}}{\sigma_{u,c}^{m}}+1-2{\left(\frac{\sigma_{u,c}^{m}-\sigma_{u,t}^{m}}{2\sigma_{u,c}^{m}}\right)}^{2}\right)\right]\right\}\times \frac{(V_{f}+0.5V_{m})E_{22}^{f}+0.5V_{m}E^{m}}{0.5E_{22}^{f}+0.5E^{m}}$$

(12) $$K_{12}=\left[1-V_{f}\frac{G_{12}^{f}-G^{m}}{G_{12}^{f}+G^{m}}\{W(V_{f})-\frac{1}{3}\}\right]\frac{(V_{f}+0.5V_{m})G_{12}^{f}+0.5V_{m}G^{m}}{0.5G_{12}^{f}+0.5G^{m}}$$

(13) $$K_{23}=2\sigma_{u,s}^{m}\sqrt{\frac{K_{22}^{t}K_{22}^{c}}{\sigma_{u,t}^{m}\sigma_{u,c}^{m}}}$$

(14) $$A=\frac{2E_{22}^{f}E^{m}{\left(\nu_{12}^{f}\right)}^{2}+E_{11}^{f}\{E^{m}(\nu_{23}^{f}-1)-E_{22}^{f}[2{\left(\nu^{m}\right)}^{2}+\nu^{m}-1]\}}{E_{11}^{f}[E_{22}^{f}+E^{m}(1-\nu_{23}^{f})+E_{22}^{f}\nu^{m}]-2E_{22}^{f}E^{m}{\left(\nu_{12}^{f}\right)}^{2}}$$

(15) $$B=\frac{E^{m}(1+\nu_{23}^{f})-E_{22}^{f}(1+\nu^{m})}{E_{22}^{f}[\nu^{m}+4{\left(\nu^{m}\right)}^{2}-3]-E^{m}(1+\nu_{23}^{f})}$$

(16) $$W(V_{f})\approx \pi \sqrt{V_{f}}[\frac{1}{4V_{f}}-\frac{4}{128}-\frac{2}{512}V_{f}-\frac{5}{4096}V_{f}{}^{2}]$$

where $\sigma_{u,t}^{m}$, $\sigma_{u,c}^{m}$ and $\sigma_{u,s}^{m}$ are the tensile, compressive and shear strengths of the matrix, respectively. $\nu_{23}^{f}$ is the fiber’s transverse Poisson’s ratio, and the other parameters are the same as those in Section 2.

In the matrix of a CFRC, no longitudinal SCF exists, because the stress field in the matrix of Figure 1a under a longitudinal load is uniform. This is, however, not true for an SFRC. Significant variation of the matrix stress would obviously occur in Figure 1b if it is subjected to a longitudinal tension. It is necessary to derive the longitudinal tensile SCF of the matrix in an SFRC.

## 4. Longitudinal Tensile SCF

**Figure 2:**
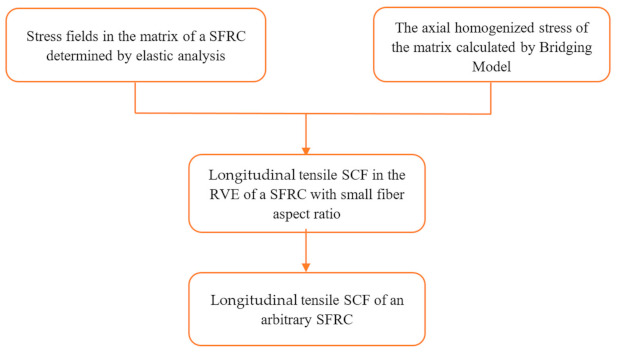
Calculation of the longitudinal tensile SCF of an SFRC.

In this section, the longitudinal tensile SCF of an SFRC is derived; the overall process of the calculation is shown in Figure 2.

According to the above general definition and Figure 1b, the longitudinal tensile SCF of the matrix in an SFRC should be given by

(17) $$K_{11}^{}=\frac{1}{L-l}\int_{l}^{L}\frac{{\tilde{\sigma }}_{11}^{m}(a,x_{1})}{{\left(\sigma_{11}^{m}\right)}_{BM}}dx_{1}$$

where *x*_1_ denotes the axial direction of the fiber, and *a* is the radius of the fiber. ${\tilde{\sigma }}_{11}^{m}$ is a point-wise stress of the matrix under a longitudinal tensile load *σ*_11_, and ${\left(\sigma_{11}^{m}\right)}_{BM}$ is the volume-averaged stress of the matrix given by the bridging model [29], i.e.,

(18) $${\left(\sigma_{11}^{m}\right)}_{BM}=\frac{a_{11}\sigma_{11}}{V_{f}+V_{m}a_{11}}=\frac{\sigma_{11}-\varepsilon_{L}^{1}E_{11}^{f}V_{f}}{1-V_{f}}$$

The first step is to determine the stress component, ${\tilde{\sigma }}_{11}^{m}(r,x_{1})$.

### 4.1. Displacements, Stresses and Strains of the Matrix

For a transversely isotropic SFRC under an axial load, the general expressions for the displacements, stresses, and strains of the matrix in RVE have already been derived in [29]. Only relevant equations are cited in this paper. The problem under study is axially symmetric; therefore, a polar coordinate system, (*z*, *r*, *θ*), is used, where *z* denotes the axial coordinate. Thus, *z* coincides with *x*_1_ in a rectangular coordinate system, as seen in Figure 1a,b. The following expressions are taken from Equations (4) and (5) and Appendix A of [29].

(19) $$u^{m(G)}=[B_{1}\sinh(nz)+B_{2}\cosh(nz)]\left\{nr[B_{3}J_{0}(nr)+{\bar{B}}_{3}Y_{0}(nr)]+[B_{4}J_{1}(nr)+{\bar{B}}_{4}Y_{1}(nr)]\right\}$$

(20) $$w^{m(G)}=-[B_{1}\cosh(nz)+B_{2}\sinh(nz)]\left\{\begin{array}{l} 4(1-\nu^{m})[B_{3}J_{0}(nr)+{\bar{B}}_{3}Y_{0}(nr)]\\ -nr[B_{3}J_{1}(nr)+{\bar{B}}_{3}Y_{1}(nr)]\\ +[B_{4}J_{0}(nr)+{\bar{B}}_{4}Y_{0}(nr)] \end{array}\right\}$$

(21) $$\sigma_{zz}^{m(G)}=-\frac{nE^{m}}{1+\nu^{m}}[B_{1}\sinh(nz)+B_{2}\cosh(nz)]\left\{\begin{array}{l} 2(2-\nu^{m})[B_{3}J_{0}(nr)+{\bar{B}}_{3}Y_{0}(nr)]\\ -nr[B_{3}J_{1}(nr)+{\bar{B}}_{3}Y_{1}(nr)]\\ +[B_{4}J_{0}(nr)+{\bar{B}}_{4}Y_{0}(nr)] \end{array}\right\}$$

(22) $$\sigma_{rr}^{m(G)}=\frac{nE^{m}}{1+\nu^{m}}[B_{1}\sinh(nz)+B_{2}\cosh(nz)]\left\{(1-2\nu^{m})[B_{3}J_{0}(nr)+{\bar{B}}_{3}Y_{0}(nr)]-\right.\;\left.nr[B_{3}J_{1}(nr)+{\bar{B}}_{3}Y_{1}(nr)]+[[B_{4}J_{0}(nr)+{\bar{B}}_{4}Y_{0}(nr)]]-\frac{1}{nr}[B_{4}J_{1}(nr)+{\bar{B}}_{4}Y_{1}(nr)]\right\}$$

(23) $$\sigma_{\theta \theta }^{m(G)}=\frac{nE^{m}}{1+\nu^{m}}[B_{1}\sinh(nz)+B_{2}\cosh(nz)]\left\{\begin{array}{l} \left(1-2\nu^{m})[B_{3}J_{0}(nr)+{\bar{B}}_{3}Y_{0}(nr)\right]\\ +\frac{1}{nr}[B_{4}J_{1}(nr)+{\bar{B}}_{4}Y_{1}(nr)] \end{array}\right\}$$

(24) $$\sigma_{rz}^{m(G)}=\frac{nE^{m}}{1+\nu^{m}}[B_{1}\cosh(nz)+B_{2}\sinh(nz)]\left\{\begin{array}{l} nr[B_{3}J_{0}(nr)+{\bar{B}}_{3}Y_{0}(nr)]\\ +2(1-\nu^{m})[B_{3}J_{1}(nr)+{\bar{B}}_{3}Y_{1}(nr)]\\ +[B_{4}J_{1}(nr)+{\bar{B}}_{4}Y_{1}(nr)] \end{array}\right\}$$

(25) $$\varepsilon_{zz}^{m(G)}=n[B_{1}\sinh(nz)+B_{2}\cosh(nz)]\left\{\begin{array}{l} -4(1-\nu^{m})[B_{3}J_{0}(nr)+{\bar{B}}_{3}Y_{0}(nr)]\\ +nr[B_{3}J_{1}(nr)+{\bar{B}}_{3}Y_{1}(nr)]\\ -[B_{4}J_{0}(nr)+{\bar{B}}_{4}Y_{0}(nr)] \end{array}\right\}$$

(26) $$\varepsilon_{rz}^{m(G)}=n[B_{1}\cosh(nz)+B_{2}\sinh(nz)]\left\{\begin{array}{l} nr[B_{3}J_{0}(nr)+{\bar{B}}_{3}Y_{0}(nr)]\\ +2(1-\nu^{m})[B_{3}J_{1}(nr)+{\bar{B}}_{3}Y_{1}(nr)]\\ +[B_{4}J_{1}(nr)+{\bar{B}}_{4}Y_{1}(nr)] \end{array}\right\}$$

The superscript “(*G*)” stands for the general solution. *u* and *w* are the displacements in the *r* and *z* directions, and *n* is an eigenvalue. *J*_0_(*nr*), *J*_1_(*nr*), *Y*_0_(*nr*), and *Y*_1_(*nr*) are Bessel functions of the first and second kinds with orders of zero and one, respectively. *B_i_* and ${\bar{B}}_{i}$ are integral constants determined by the boundary and continuity conditions applied on the RVE. Note that in the fiber end region Ω_2_, all of the quantities except for the material properties should have a tilde “∼” overhead.

### 4.2. Determination of the Constants in Ω_1_

In Ω_1_, the boundary conditions are given by [29]

(27) $$w^{m(G)}(r,0)=0,\tau_{rz}^{m(G)}(r,0)=0,\tau_{rz}^{m(G)}(b,z)=0,u^{m(G)}(b,z)=0$$

where *b* is the radius of the matrix. Based on Equation (27), one can obtain

*B*_1_ = 0 (28)

(29) $$B_{3}J_{1}(nb)+{\bar{B}}_{3}Y_{1}(nb)=0$$

(30) $$nb[B_{3}J_{0}(nb)+{\bar{B}}_{3}Y_{0}(nb)]+[B_{4}J_{1}(nb)+{\bar{B}}_{4}Y_{1}(nb)]=0$$

By use of a shear-lag condition, this leads to

(31) $$\frac{d\sigma_{zz}^{f(G)}}{dz}=-\frac{2\tau_{rz}^{m(G)}(a,z)}{a}=-\frac{2}{a}\frac{nE^{m}B_{2}\sinh(nz)}{\left(1+\nu^{m}\right)}\left\{na[B_{3}J_{0}(na)+{\bar{B}}_{3}Y_{0}(na)]\right.\;\left.+2(1-\nu^{m})[B_{3}J_{1}(na)+{\bar{B}}_{3}Y_{1}(na)]+[B_{4}J_{1}(na)+{\bar{B}}_{4}Y_{1}(na)]\right\}$$

By integrating with respect to *z* and using the stress balance condition along the longitudinal direction, one obtains

(32) $$\sigma_{zz}^{f(G)}(a,z)=-\frac{2}{a}\frac{nE^{m}B_{2}cosh(nz)}{n(1+\nu^{m})}\left\{na[B_{3}J_{0}(na)+{\bar{B}}_{3}Y_{0}(na)]\right.\;\left.+2(1-\nu^{m})[B_{3}J_{1}(na)+{\bar{B}}_{3}Y_{1}(na)]+[B_{4}J_{1}(na)+{\bar{B}}_{4}Y_{1}(na)]\right\}$$

At the interface between the fiber and matrix, the following continuity conditions are applied:

(33) $$\varepsilon_{zz}^{f(G)}(a,z)=\varepsilon_{zz}^{m(G)}(a,z),\varepsilon_{\theta \theta }^{f(G)}(a,z)=\varepsilon_{\theta \theta }^{m(G)}(a,z),\sigma_{rr}^{f(G)}(a,z)=\sigma_{rr}^{m(G)}(a,z)$$

Based on Equation (33) and the constitutive equations of fiber and matrix, it can be derived that

(34) $$\sigma_{zz}^{f(G)}(a,z)=\frac{E_{11}^{f}}{[E_{11}^{f}-E_{22}^{f}{\left(\upsilon_{12}^{f}\right)}^{2}]E^{m}}\left[\begin{array}{l} \left(E_{11}^{f}-\nu^{m}\nu_{12}^{f}E_{22}^{f})\sigma_{zz}^{m(G)}(a,z)+[E^{m}\nu_{12}^{f}(1+\nu_{23}^{f}\right)\\ -\nu^{m}(E_{11}^{f}+\nu_{12}^{f}E_{22}^{f})]\sigma_{rr}^{m(G)}(a,z)\\ +(\nu_{12}^{f}E_{22}^{f}-\nu^{m}E_{11}^{f})\sigma_{\theta \theta }^{m(G)}(a,z) \end{array}\right]$$

Substituting Equations (21)–(23) into the last equation leads to

(35) $$\sigma_{zz}^{f(G)}(a,z)=\frac{nB_{2}E_{11}^{f}\cosh(nz)}{\left[E_{11}^{f}-E_{22}^{f}{\left(\nu_{12}^{f}\right)}^{2}](1+\nu^{m}\right)}\left((\nu^{m}\nu_{12}^{f}E_{22}^{f}-E_{11}^{f})\left\{\left[B_{4}J_{0}(na)+{\bar{B}}_{4}Y_{0}(na)\right]\right.\right.\;\left.-na[B_{3}J_{1}(na)+{\bar{B}}_{3}Y_{1}(na)]+2(2-\nu^{m})[B_{3}J_{0}(na)+{\bar{B}}_{3}Y_{0}(na)]\right\}\;+[E^{m}\nu_{12}^{f}(1+\nu_{23}^{f})-\nu^{m}(E_{11}^{f}+\nu_{12}^{f}E_{22}^{f})]\left\{\left(1-2\nu^{m}\right)\left[B_{3}J_{0}(na)+{\bar{B}}_{3}Y_{0}(na)\right]\right.\;-na\left[B_{3}J_{1}(na)+{\bar{B}}_{3}Y_{1}(na)\right]+\left[B_{4}J_{0}(na)+{\bar{B}}_{4}Y_{0}(na)\right]\;\left.-\frac{1}{na}\left[B_{4}J_{1}(na)+{\bar{B}}_{4}Y_{1}(na)\right]\right\}+(\nu_{12}^{f}E_{22}^{f}-\nu^{m}E_{11}^{f})\left\{\frac{1}{na}\left[B_{4}J_{1}(na)+{\bar{B}}_{4}Y_{1}(na)\right]\right.\;\left.+(1-2\nu^{m})\left[B_{3}J_{0}(na)+{\bar{B}}_{3}Y_{0}(na)\right]\right\}$$

Another continuity condition (refer to Equation (17) of [29]) reads

(36) $$\sigma_{zz}^{f(G)}(a,z)=E_{11}^{f}\varepsilon_{zz}^{m(G)}(a,z)+2\nu_{12}^{f}\sigma_{rr}^{m(G)}(a,z)$$

(37) $$\begin{array}{l} \sigma_{zz}^{f(G)}(a,z)=E_{11}^{f}nB_{2}\cosh(nz)\left\{-4(1-\nu^{m})[B_{3}J_{0}(na)+{\bar{B}}_{3}Y_{0}(na)]+\right.\\ \left.+na[B_{3}J_{1}(na)+{\bar{B}}_{3}Y_{1}(na)]-[B_{4}J_{0}(na)+{\bar{B}}_{4}Y_{0}(na)]\right\}+\frac{2\nu_{12}^{f}E^{m}nB_{2}\cosh(nz)}{\left(1+\nu^{m}\right)} \end{array}\;\times \left\{\left(1-2\nu^{m})[B_{3}J_{0}(na)+{\bar{B}}_{3}Y_{0}(na)]-na[B_{3}J_{1}(na)+{\bar{B}}_{3}Y_{1}(na)\right]\right.\;\left.+[B_{4}J_{0}(na)+{\bar{B}}_{4}Y_{0}(na)]-\frac{1}{na}[B_{4}J_{1}(na)+{\bar{B}}_{4}Y_{1}(na)]\right\}$$

Letting Equation (32) = Equation (35) and Equation (32) = Equation (37) and using Equation (29), one obtains that

(38) $$B_{4}=\frac{Y_{1}(na)\left(\alpha \beta_{2}-\alpha_{2}\beta \right)+Y_{0}(na)\left(\alpha \beta_{1}-\alpha_{1}\beta \right)}{\left(\alpha_{1}\beta_{2}-\alpha_{2}\beta_{1}\right)\left[J_{0}(na)Y_{1}(na)-Y_{0}(na)J_{1}(na)\right]}$$

(39) $${\bar{B}}_{4}=-\frac{J_{1}(na)\left(\alpha \beta_{2}-\alpha_{2}\beta \right)+J_{0}(na)\left(\alpha \beta_{1}-\alpha_{1}\beta \right)}{\left(\alpha_{1}\beta_{2}-\alpha_{2}\beta_{1}\right)\left[J_{0}(na)Y_{1}(na)-Y_{0}(na)J_{1}(na)\right]}$$

(40) $$\alpha_{1}=\frac{nE_{11}^{f}\left[\nu_{12}^{f}E^{m}(1+\nu_{23}^{f})-E_{11}^{f}(1+\nu^{m})\right]}{E_{11}^{f}-E_{22}^{f}{\left(\nu_{12}^{f}\right)}^{2}}$$

(41) $$\alpha_{2}=\frac{2E^{m}}{a}+\frac{E_{11}^{f}\left[\nu_{12}^{f}E_{22}^{f}(1+\nu^{m})-\nu_{12}^{f}E^{m}(1+\nu_{23}^{f})\right]}{a\left[E_{11}^{f}-E_{22}^{f}{\left(\nu_{12}^{f}\right)}^{2}\right]}$$

(42) $$\beta_{1}=\frac{2\nu_{12}^{f}E^{m}}{1+\nu^{m}}-E_{11}^{f}$$

(43) $$\beta_{2}=\frac{2E^{m}\left(1-\nu_{12}^{f}\right)}{na(1+\nu^{m})}$$

(44) $$\alpha =\left\{\frac{nE_{11}^{f}\left\{\begin{array}{l} (2\nu^{m}-1)\left[\nu_{12}^{f}E_{22}^{f}(1-\nu^{m})-2\nu^{m}E_{11}^{f}+\nu_{12}^{f}E^{m}(1+\nu_{23}^{f})\right]\\ -(2\nu^{m}-4)\left[E_{11}^{f}-\nu^{m}\nu_{12}^{f}E^{m}E_{22}^{f}\right] \end{array}\right\}}{E_{11}^{f}-E_{22}^{f}{\left(\nu_{12}^{f}\right)}^{2}}-2nE^{m}\right\}\times \;[B_{3}J_{0}(na)+{\bar{B}}_{3}Y_{0}(na)]+\left\{\frac{4E^{m}(\nu^{m}-1)}{a}+\frac{n^{2}aE_{11}^{f}\left[\nu_{12}^{f}E^{m}(1+\nu_{23}^{f})-E_{11}^{f}(1+\nu^{m})\right]}{E_{11}^{f}-E_{22}^{f}{\left(\nu_{12}^{f}\right)}^{2}}\right\}\;\times [B_{3}J_{1}(na)+{\bar{B}}_{3}Y_{1}(na)]$$

(45) $$\begin{array}{l} \beta =\left\{\frac{2E^{m}\left[\nu_{12}^{f}(2\nu^{m}-1)-1\right]}{1+\nu^{m}}+4E_{11}^{f}(1-\nu^{m})\right\}\times [B_{3}J_{0}(na)+{\bar{B}}_{3}Y_{0}(na)]\\ +\left\{\frac{2E^{m}\left[n^{2}a^{2}\nu_{12}^{f}-2(1-\nu^{m})\right]}{na(1+\nu^{m})}-naE_{11}^{f}\right\}\times [B_{3}J_{1}(na)+{\bar{B}}_{3}Y_{1}(na)] \end{array}$$

where *n* is the smallest but positive root to Equation (30).

### 4.3. Determination of the Constants in Ω_2_

In Ω_2_, assuming that an imaginary fiber with a radius of *a* is contained, properties of the fiber are the same as those of the matrix. Using the same boundary conditions, continuity conditions and solution methods as in Ω_1_, one obtains

(46) $${\tilde{B}}_{3}=0,\;{\tilde{\bar{B}}}_{3}=0$$

(47) $${\tilde{B}}_{1}=-{\tilde{B}}_{2}\frac{\sinh(\tilde{n}L)}{\cosh(\tilde{n}L)}$$

(48) $${\tilde{\bar{B}}}_{4}=-{\tilde{B}}_{4}\frac{J_{1}(\tilde{n}b)}{Y_{1}(\tilde{n}b)}$$

(49) $$\left[\tilde{n}aJ_{0}(\tilde{n}a)-2J_{1}(\tilde{n}a)\right]Y_{1}(\tilde{n}b)-\left[\tilde{n}aY_{0}(\tilde{n}a)-2Y_{1}(\tilde{n}a)\right]J_{1}(\tilde{n}b)=0$$

The quantities with ∼ overhead denote those in Ω_2_. $\tilde{n}$ is the smallest positive root to Equation (49).

### 4.4. Continuity Conditions in between Ω_1_ and Ω_2_

In the above, two combined constants, *B*_2_*B*_3_ and ${\tilde{B}}_{2}{\tilde{B}}_{4}$, remain to be determined.

Simplifying the relevant equations in Ω_1_, one can obtain

(50) $$\sigma_{zz}^{f}(a,z)=nD\cosh(nz)\xi_{f}+\sigma_{zz}^{f(S)}(a,z)$$

(51) $$\sigma_{rz}^{m(G)}(a,z)=nD\sinh(nz)\xi_{rz}$$

(52) $$\xi_{f}=-\frac{2E^{m}}{na(1+\nu^{m})}\left\{\begin{array}{l} na[D_{1}J_{0}(na)+D_{2}Y_{0}(na)]+2(1-\nu^{m})[D_{1}J_{1}(na)+D_{2}Y_{1}(na)]\\ +[D_{3}J_{1}(na)+D_{4}Y_{1}(na)] \end{array}\right\}$$

(53) $$\xi_{rz}=\frac{E^{m}}{1+\nu^{m}}\left\{\begin{array}{l} na[D_{1}J_{0}(na)+D_{2}Y_{0}(na)]+2(1-\nu^{m})[D_{1}J_{1}(na)+D_{2}Y_{1}(na)]\\ +[D_{3}J_{1}(na)+D_{4}Y_{1}(na)] \end{array}\right\}$$

(54) $$D=B_{2}B_{3},\;D_{1}=B_{3}/B_{3}=1,\;D_{2}={\bar{B}}_{3}/B_{3},\;D_{3}=B_{4}/B_{3},\;D_{4}={\bar{B}}_{4}/B_{3}$$

The superscript “(*S*)” stands for the specific solution. In Equation (50), $\sigma_{zz}^{f(S)}(a,z)$ is determined by Equation (55) [29].

(55) $$\sigma_{zz}^{f(S)}(a,z)=E_{11}^{f}\varepsilon_{L}^{1}+\frac{2\upsilon_{12}^{f}E^{m}}{\left(1+\nu^{m})(1-2\nu^{m}\right)}\left[\varepsilon_{L}^{1}\nu^{m}+C_{1}-C_{2}\frac{1-2\nu^{m}}{a^{2}}\right]$$

Additionally, the relevant stresses in Ω_2_ can be simplified as

(56) $${\tilde{\sigma }}_{zz}^{f}(a,z)=\tilde{n}\tilde{D}\cosh\left[\tilde{n}(z-L)\right]{\tilde{\xi }}_{f}+{\tilde{\sigma }}_{zz}^{f(S)}(a,z)$$

(57) $${\tilde{\sigma }}_{rz}^{m(G)}(a,z)=\tilde{n}\tilde{D}\sinh[\tilde{n}(z-L)]{\tilde{\xi }}_{rz}$$

(58) $${\tilde{\xi }}_{f}=-\frac{2E^{m}}{\tilde{n}a(1+\nu^{m})}[{\tilde{D}}_{1}J_{1}(\tilde{n}a)+{\tilde{D}}_{2}Y_{1}(\tilde{n}a)]$$

(59) $${\tilde{\xi }}_{rz}=\frac{E^{m}}{1+\nu^{m}}[{\tilde{D}}_{1}J_{1}(\tilde{n}a)+{\tilde{D}}_{2}Y_{1}(\tilde{n}a)]$$

(60) $$\tilde{D}=\frac{{\tilde{B}}_{2}{\tilde{B}}_{4}}{\cosh(\tilde{n}L)},\;\tilde{D}={\tilde{B}}_{4}/{\tilde{B}}_{4}=1,\;{\tilde{D}}_{2}={\tilde{\bar{B}}}_{4}/{\tilde{B}}_{4}=-\frac{J_{1}(\tilde{n}b)}{Y_{1}(\tilde{n}b)}$$

${\tilde{\sigma }}_{zz}^{f(S)}(a,z)$ in Equation (56) is given by [29]

(61) $${\tilde{\sigma }}_{zz}^{f(S)}(a,z)=\frac{E^{m}}{\left(1+\nu^{m})(1-2\nu^{m}\right)}\left[\varepsilon_{L}^{2}(1-\nu^{m})+\frac{(1+\nu^{m})(1-2\nu^{m})\sigma_{11}-(1-\nu^{m})E^{m}\varepsilon_{L}^{2}}{E^{m}}\right]$$

Based on the continuity conditions between Ω_1_ and Ω_2_

(62) $$\sigma_{rz}^{m(G)}(a,l)={\tilde{\sigma }}_{rz}^{m(G)}(a,l),\sigma_{zz}^{f}(a,l)={\tilde{\sigma }}_{zz}^{f}(a,l)=\sigma_{FD}$$

One derives that,

(63) $$nD=\frac{\sigma_{FD}-\sigma_{zz}^{f(S)}(a,l)}{\cosh(nl)\xi_{f}}$$

(64) $$\tilde{n}\tilde{D}=\frac{\sigma_{FD}-{\tilde{\sigma }}_{zz}^{f(S)}(a,l)}{\cosh[\tilde{n}(l-L)]{\tilde{\xi }}_{f}}$$

(65) $$\sigma_{FD}=\frac{{\bar{\sigma }}_{zz}^{f(S)}\xi_{rz}{\tilde{\xi }}_{f}\tanh(nl)-{\bar{\tilde{\sigma }}}_{zz}^{f(S)}{\tilde{\xi }}_{rz}\xi_{f}\tanh[\tilde{n}(l-L)]}{\xi_{rz}{\tilde{\xi }}_{f}\tanh(nl)-{\tilde{\xi }}_{rz}\xi_{f}\tanh[\tilde{n}(l-L)]}$$

where ${\bar{\sigma }}_{zz}^{f(S)}$ and ${\bar{\tilde{\sigma }}}_{zz}^{f(S)}$ are the same as Equations (55) and (61), respectively. $\varepsilon_{L}^{1}$ and $\varepsilon_{L}^{2}$ are the same as those in Equation (6). These, together with *C*_1_ and *C*_2_ in Equation (55), are obtained from solving equations in Appendix A.

### 4.5. Integration for Equation (17)

It should be mentioned that the elastic solution to ${\tilde{\sigma }}_{11}^{m}\left(r,x_{1}\right)$ is superposed from general and specific solutions. Thus, in Equation (17)

(66) $${\tilde{\sigma }}_{11}^{m}\left(a,x_{1}\right)\equiv {\tilde{\sigma }}_{zz}^{m}\left(a,z\right)={\tilde{\sigma }}_{zz}^{m(S)}\left(a,z\right)+{\tilde{\sigma }}_{zz}^{m(G)}\left(a,z\right)$$

The specific solution is obtained from Equations (21) and (24) of [29] as

(67) $${\tilde{\sigma }}_{{}_{zz}}^{m(S)}=\frac{E^{m}}{\left(1+\nu^{m})(1-2\nu^{m}\right)}\left[2{\tilde{C}}_{1}\nu^{m}+\varepsilon_{L}^{2}(1-\nu^{m})\right]$$

(68) $${\tilde{C}}_{1}=\frac{(1+\nu^{m})(1-2\nu^{m})\sigma_{11}-(1-\nu^{m})E^{m}\varepsilon_{L}^{2}}{2\nu^{m}E^{m}}$$

whereas the general solution is given by

(69) $${\tilde{\sigma }}_{zz}^{m(G)}\left(a,z\right)=-\frac{\tilde{n}\tilde{D}E^{m}\cosh\left[\tilde{n}\left(z-L\right)\right]}{1+\nu^{m}}\left[J_{0}\left(\tilde{n}a\right)-\frac{J_{1}\left(\tilde{n}b\right)}{Y_{1}\left(\tilde{n}b\right)}Y_{0}\left(\tilde{n}a\right)\right]$$

$\tilde{n}\tilde{D}$ is determined by Equation (64).

Substituting Equations (18), (67) and (69) into Equation (17), one finds that

(70) $$K_{11}^{I}=\frac{(1-V_{f})E^{m}}{\sigma_{11}-\varepsilon_{L}^{1}E_{11}^{f}V_{f}}\left\{\frac{\sigma_{11}}{E^{m}}-\frac{\tilde{D}\sinh[\tilde{n}(L-l)][J_{0}(\tilde{n}a)-\frac{J_{1}(\tilde{n}b)}{Y_{1}(\tilde{n}b)}Y_{0}(\tilde{n}a)]}{\left(1+\nu^{m})(L-l\right)}\right\}$$

The superscript *I* denotes that the SCF is obtained from Equation (17).

It can be found by calculation that when the fiber aspect ratio, *l*/*a*, tends to infinity, the SCF determined by Equation (70) becomes very large. In other words, when the SFRC becomes a CFRC, the longitudinal tensile SCF given by Equation (70) cannot deteriorate to unity. Thus, the SCF defined by Equation (70) must be modified to make it applicable to an SFRC with an arbitrary fiber aspect ratio.

### 4.6. Derivation of K_11_

**Figure 3:**
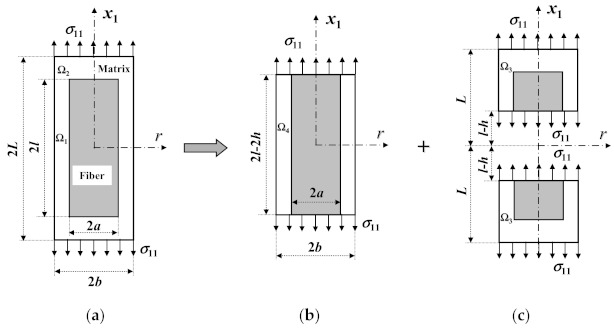
(**a**) RVE of an SFRC; (**b**) central segment; (**c**) end segments.

Considering this, the RVE of an SFRC is subdivided into three segments, namely, a central segment and two ends, as shown in Figure 3. According to the condition that the true longitudinal matrix stress in Figure 3a equals that in Figure 3b plus that in Figure 3c, the following equation is obtained

(71) $$2LK_{11}^{}{\left(\sigma_{11}^{m}\right)}_{BM}=2(L-l+h)K_{11}^{II}{\left(\sigma_{11}^{m}\right)}_{av}^{II}+(2l-2h)\frac{E^{m}}{TE_{11}^{f}+(1-T)E^{m}}\sigma_{11},\;\mathrm{for}\;l\;>\;h$$

(72) $$K_{11}^{II}=\frac{1}{L-l}\int_{l}^{L}\frac{{\tilde{\sigma }}_{11}^{m}(a,x_{1})}{{\left(\sigma_{11}^{m}\right)}_{av}^{II}}dx_{1},\;{\left(\sigma_{11}^{m}\right)}_{av}^{II}=\frac{\int_{{}_{\Omega_{3}}}{\tilde{\sigma }}_{11}^{m}(r,x_{1})dV}{\pi b^{2}(L-l)+\pi (b^{2}-a^{2})h}$$

*T* = (*a/b*)^2^ (73)

${\left(\sigma_{11}^{m}\right)}_{BM}$ is the same as Equation (18), ${\tilde{\sigma }}_{11}^{m}\left(r,x_{1}\right)$ can be obtained from Equation (66), and *h* is the fiber length in the end segment Ω_3_.

It can be seen that Figure 3b is the same as Figure 1a, and no matrix’s SCF in the longitudinal direction exists. By using Equations (17), (18), (71) and (72), the longitudinal tensile SCF, *K*_11_, is derived as

(74) $$K_{11}^{}=\left\{\begin{array}{c} K_{11}^{I},\;if\;l\leq h,\\ \frac{L-l+h}{L}K_{11}^{I}+\frac{l-h}{L}\frac{V_{m}E^{m}\sigma_{11}^{}}{\left(\sigma_{11}^{}-\varepsilon_{L}^{1}E_{11}^{f}V_{f})(TE_{11}^{f}+(1-T)E^{m}\right)},if\;l>h \end{array}\right.$$

To calculate *K*_11_ by Equation (74), the parameter *h* needs to be determined. Considering the axial stress balance at any cross-section and to mostly resemble a CFRC under axial tension, the distribution of stress on any cross-section of the RVE should be near to uniform. The averaged axial stress of the matrix in a cross-section near to the fiber end (*z* = *l*) is higher than that at a central cross-section; therefore, we may impose that the averaged fiber axial stress is equal to 0.99 ${\bar{\sigma }}_{zz}^{f}(0)$, where ${\bar{\sigma }}_{zz}^{f}(0)$ is the averaged fiber axial stress at the central cross-section of Figure 3a. The averaged fiber axial stress in Ω_1_ can be obtained from Equation (37) of [38] as

(75) $${\bar{\sigma }}_{zz}^{f}=\left(\sigma_{FD}-{\bar{\sigma }}_{zz}^{f\left(S\right)}\right)\frac{\cosh(nz)}{\cosh(nl)}+{\bar{\sigma }}_{zz}^{f\left(S\right)}$$

$\sigma_{FD}$ is determined by Equation (62), and ${\bar{\sigma }}_{zz}^{f(S)}$ is the same as Equation (51).

It can be easily found that when the parameter *nl* is sufficiently large, the denominator will be infinite, and it is unsolvable for *z* = *l − h*. Through numerical experiments with *Mathematica*, we found that the maximum solvable aspect ratio is near to *l/a* = 200. Therefore, we assume that *h* is a solution to one of the following equations:

(76) $$[\sigma_{FD}-{\bar{\sigma }}_{zz}^{f(S)}]\frac{\cosh[n(l-h)]}{\cosh(nl)}+{\bar{\sigma }}_{zz}^{f(S)}=0.99{\bar{\sigma }}_{zz}^{f}(0),\;\mathrm{if}\;l/a\;\leq \;200$$

(77) $$[\sigma_{FD}-{\bar{\sigma }}_{zz}^{f(S)}]\frac{\cosh[n(l-h)]}{\cosh(200na)}+{\bar{\sigma }}_{zz}^{f(S)}=0.99{\bar{\sigma }}_{zz}^{f}(0),\;\mathrm{if}\;l/a\;>\;200$$

(78) $${\bar{\sigma }}_{zz}^{f}(0)=[\sigma_{FD}-{\bar{\sigma }}_{zz}^{f(S)}]/\cosh(nl)+{\bar{\sigma }}_{zz}^{f(S)}$$

**Figure 4:**
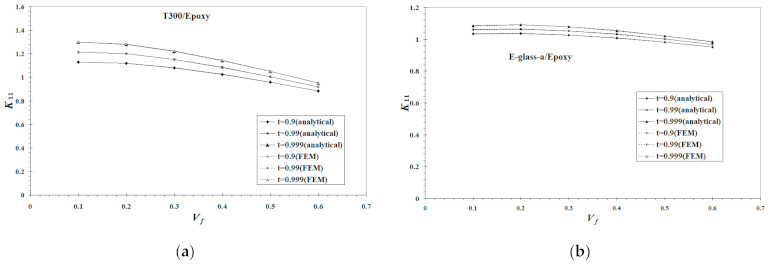
Influence of the chosen *h*’s through $t={\bar{\sigma }}_{zz}^{f}(l-h)/{\bar{\sigma }}_{zz}^{f}(0)$ on longitudinal tensile SCF of the matrix in (**a**) T300/epoxy and (**b**) E-glass-a/epoxy systems.

Although the *h* obtained from Equations (76) and (77) is somewhat arbitrary, a comparison study on different *h* values shown in Figure 4 by setting ${\bar{\sigma }}_{zz}^{f}(l-h)$ to a value in between $0.9{\bar{\sigma }}_{zz}^{f}(0)$ and $0.999{\bar{\sigma }}_{zz}^{f}(0)$ indicates that the resulting *K*_11_ is close to each other. Considering that truncation errors are inevitable in calculating a value from the many transcendental functions involved in this work, the present choice is pertinent.

## 5. Failure Criterion and Strength Prediction

### 5.1. Failure Criterion

In this paper, the fiber and matrix are assumed to be bonded perfectly, and a composite failure is attained when any failure of the fiber or matrix is assumed. The corresponding load is defined as a composite strength. If, however, an interface crack between the fiber and matrix exists, one has to consider the usage of the matrix’s SCF after the interface crack, following [28].

The fiber is thin, like a bar, and the generalized maximum normal stress failure criterion [34] is well applicable to detect a fiber failure, as per

(79) $$\sigma_{eq,t}^{f}\geq \sigma_{u,t}^{f}$$

(80) $$\sigma_{eq,t}^{f}=\left\{\begin{array}{c} \sigma_{f}^{1},\;if\;\sigma_{f}^{3}<0\\ {\left[{\left(\sigma_{f}^{1}\right)}^{3}+{\left(\sigma_{f}^{2}\right)}^{3}\right]}^{1/3},\;if\;\sigma_{f}^{3}=0\\ {\left[{\left(\sigma_{f}^{1}\right)}^{3}+{\left(\sigma_{f}^{2}\right)}^{3}+{\left(\sigma_{f}^{3}\right)}^{3}\right]}^{1/3},\;if\;\sigma_{f}^{3}>0 \end{array}\right.$$

where $\sigma_{f}^{1}$, $\sigma_{f}^{2}$ and $\sigma_{f}^{3}$ are the three principal stresses of the fiber, and $\sigma_{u,t}^{f}$ is its longitudinal tensile strength.

Once the conversion from the homogenized into the true stresses is made, a matrix failure can be detected as if there were no fiber reinforcement. In this study, the Tsai–Wu criterion [34] was used to detect a matrix failure,

(81) $$F_{1}({\bar{\sigma }}_{11}^{m}+{\bar{\sigma }}_{22}^{m}+{\bar{\sigma }}_{33}^{m})+F_{11}[{\left({\bar{\sigma }}_{11}^{m}\right)}^{2}+{\left({\bar{\sigma }}_{22}^{m}\right)}^{2}+{\left({\bar{\sigma }}_{33}^{m}\right)}^{2}-{\bar{\sigma }}_{11}^{m}{\bar{\sigma }}_{22}^{m}]-\;F_{11}({\bar{\sigma }}_{11}^{m}{\bar{\sigma }}_{33}^{m}+{\bar{\sigma }}_{22}^{m}{\bar{\sigma }}_{33}^{m})+F_{44}[{\left({\bar{\sigma }}_{23}^{m}\right)}^{2}+{\left({\bar{\sigma }}_{13}^{m}\right)}^{2}+{\left({\bar{\sigma }}_{12}^{m}\right)}^{2}]\geq 1$$

(82) $$F_{1}=\frac{1}{\sigma_{u,t}^{m}}-\frac{1}{\sigma_{u,c}^{m}},\;F_{11}=\frac{1}{\sigma_{u,t}^{m}\sigma_{u,c}^{m}},\;F_{44}=\frac{1}{{\left(\sigma_{u,c}^{m}\right)}^{2}}$$

(83) $$\{{\bar{\sigma }}_{i}^{m}\}={\left\{K_{11}^{}\sigma_{11}^{m},K_{2}\sigma_{22}^{m},K_{3}\sigma_{33}^{m},K_{23}\sigma_{23}^{m},K_{12}\sigma_{13}^{m},K_{12}\sigma_{12}^{m}\right\}}_{\sigma_{22}^{m}\times \sigma_{33}^{m}=0}{}_{\;}$$

(84) $$K_{2}=\left\{\begin{array}{l} K_{22}^{t},if^{}\sigma_{33}^{m}=0_{}{\&}_{}\sigma_{22}^{m}>0\\ K_{22}^{c},if^{}\sigma_{33}^{m}=0_{}{\&}_{}\sigma_{22}^{m}<0 \end{array}\right.$$

(85) $$K_{3}=\left\{\begin{array}{l} K_{22}^{t},if^{}\sigma_{22}^{m}=0_{}{\&}_{}\sigma_{33}^{m}>0\\ K_{22}^{c},if^{}\sigma_{22}^{m}=0_{}{\&}_{}\sigma_{33}^{m}<0 \end{array}\right.$$

$\sigma_{u,t}^{m}$, $\sigma_{u,c}^{m}$, and $\sigma_{u,s}^{m}$ are the tensile, compressive, and shear strengths of the matrix, respectively, which can be measured using the monolithic material coupons. The homogenized stresses $\left\{\sigma_{i}^{m}\right\}$ are calculated by the bridging model, and stresses with “-” overhead represent true stresses. The calculation of $K_{22}^{t}$,$K_{22}^{c}$,$K_{12}^{}$, and $K_{23}$ can be obtained from Equations (10)–(13), $K_{11}^{}$ is determined by Equation (74).

### 5.2. Strength of UA Short Fiber Composites

A UA (uniaxially aligned) short fiber composite to an SFRC is of the same importance as a UD lamina to a CFRC. Any SFRC can be regarded as a combination of a series of UA short fiber composites.

Under a longitudinal tensile load, the composite strength $\sigma_{11}^{u,t}$ is assumed when either of the following conditions is attained [34]

(86) $$\sigma_{11}^{f}=\frac{\sigma_{11}^{u,t}}{V_{f}+V_{m}a_{11}}\geq \sigma_{u,t}^{f},\;{\bar{\sigma }}_{11}^{m}=\frac{K_{11}^{}a_{11}\sigma_{11}^{u,t}}{V_{f}+V_{m}a_{11}}\geq \sigma_{u,t}^{m}$$

Thus, the strength formula is expressed as

(87) $$\sigma_{11}^{u,t}=\min\left\{\sigma_{u,t}^{f}\left(V_{f}+V_{m}a_{11}\right),\frac{V_{f}+V_{m}a_{11}}{a_{11}}\frac{\sigma_{u,t}^{m}}{K_{11}^{}}\right\}$$

where *a*_11_ refers to Equation (6) and $K_{11}^{}$ is given by Equation (74).

### 5.3. Strength of Randomly Oriented Short Fiber Composite

The analysis of a randomly oriented SFRC additionally needs a subdivision and an assemblage. The subdivision of the SFRC into a series of elements is carried out, so that each element contains only one fiber segment, which can be regarded as a UA SFRC. An iso-stress assemblage is applied, which states that each element (the *k*th element) sustains a global load the same as that applied on the composite, i.e.,

(88) $$\{\sigma_{i}^{G}\}={\left\{\sigma_{i}^{G}\right\}}_{k}$$

*G* represents the global coordinate system. The global load can be converted into the local load through [34]

(89) $${\left\{\sigma_{i}\right\}}_{k}={\left[T_{ij}^{k}\right]}_{s}^{T}{\left\{\sigma_{j}^{G}\right\}}_{k}$$

where ${\left[T_{ij}^{k}\right]}_{s}^{}$ is a coordinate transformation tensor given in Appendix B, and the superscript *T* denotes a transpose. Substituting the element local stresses from Equation (89) into Equations (4) and (5), the homogenized stresses of the fiber and matrix in the element can be determined. They are transformed into the global coordinate system as per

(90) $${\left\{{\bar{\sigma }}_{i}^{f}\right\}}_{k}^{G}={\left[T_{ij}^{k}\right]}_{c}{\left\{{\bar{\sigma }}_{i}^{f}\right\}}_{k}$$

(91) $${\left\{{\bar{\sigma }}_{i}^{m}\right\}}_{k}^{G}={\left[T_{ij}^{k}\right]}_{c}{\left\{{\bar{\sigma }}_{i}^{m}\right\}}_{k}$$

(92) $${\left\{{\bar{\sigma }}_{i}^{f}\right\}}_{k}\equiv {\left\{\sigma_{i}^{f}\right\}}_{k}={\left(V_{f}[I]+V_{m}[a_{ij}]\right)}^{-1}{\left[T_{ij}^{k}\right]}_{s}^{T}{\left\{\sigma_{j}^{G}\right\}}_{k}$$

(93) $${\left\{\sigma_{i}^{m}\right\}}_{k}=[a_{ij}]{\left(V_{f}[I]+V_{m}[a_{ij}]\right)}^{-1}{\left[T_{ij}^{k}\right]}_{s}^{T}{\left\{\sigma_{j}^{G}\right\}}_{k}$$

${\left[T_{ij}^{k}\right]}_{c}$ is another coordinate transformation tensor given in Appendix B. ${\left\{{\bar{\sigma }}_{i}^{m}\right\}}_{k}$ is the same as Equation (83). The true stresses of the fiber and matrix in the composite in the global system can be obtained through a superposition

(94) $${\left\{{\bar{\sigma }}_{i}^{f}\right\}}^{G}=\sum_{k}f(\theta_{k}){\left\{{\bar{\sigma }}_{i}^{f}\right\}}_{k}^{G}$$

(95) $${\left\{{\bar{\sigma }}_{i}^{m}\right\}}^{G}=\sum_{k}f(\theta_{k}){\left\{{\bar{\sigma }}_{i}^{m}\right\}}_{k}^{G}$$

*f*(*θ*) is a fiber orientation distribution function, which is taken as [39]

(96) $$f(\theta )=\frac{\lambda e^{-\lambda \theta }}{1-e^{-\frac{\pi }{2}\lambda }},0\leq \theta \leq \frac{\pi }{2}$$

where *θ* is the orientation angle of the fiber. *λ* is a shape parameter reflecting the fiber orientation extent. A smaller value indicates a more random orientation, e.g., *λ* = 1 and 100 represent randomly oriented and highly aligned fibers, respectively [40].

## 6. Numerical Examples

### 6.1. Fiber Length Ratio

In order to predict the tensile strength of an SFRC, except for the fiber aspect ratio *ξ = l/a* and the fiber volume fraction *V_f_*, the fiber length ratio *γ = L*/*l* in the RVE must be provided as well. There is no method to directly measure *γ*, and an empirical formula proposed in [40] is used, i.e.,

(97) $$\gamma =\frac{1}{{\left(\sqrt{V_{f}}\xi \tan\left(\arctan(1/\xi )-\frac{\arctan(1/\xi )-\arctan(1/(\xi \sqrt{V_{f}}))}{g(\xi )}\right)\right)}^{2/3}}$$

(98) $$g\left(\xi \right)=\frac{1}{c_{0}(\xi -1)}+1$$

*c*_0_ = 0.03 was found to be pertinent from the correlation between the predicted and measured elastic moduli of several SFRCs, and is used in this work.

### 6.2. Material Parameters

To verify our theory, the predicted results were compared with the available experimental data. Three fibers together with two polymer matrices were chosen; the material properties are given in Table 2 and the geometric parameters are shown in Table 3. According to the data in , the matrices’ SCFs can be calculated, and are also shown in Table 3. It can easily be found that when the fiber aspect ratio is large enough, e.g., *ξ* = 429, the longitudinal tensile SCF is always near to unity no matter what the fiber volume fraction is; if the fiber aspect ratio is moderate, e.g., *ξ* = 25, the longitudinal tensile SCF noticeably differs from unity.

### 6.3. Results

#### 6.3.1. *K*_11_

In addition to the constituent material and geometric parameters, the matrix longitudinal tensile SCF, *K*_11_, may be influenced to some extent by the length of a fiber end segment, *h*. Furthermore, different choices for the RVE geometries for an SFRC together with varied boundary conditions may also result in a variation of the obtained *K*_11_. In this paper, a CCA (concentric cylinder assemblage) model together with infinity boundary conditions was chosen for the RVE geometry so that exact elastic solutions were available. In [35], a finite volume RVE containing a single short fiber specified with periodic boundary conditions was adopted. A numerical comparison study for all these different *K*_11_ values was carried out, and is plotted in Figure 4. Two material systems, i.e., T300/epoxy and E-glass-a/epoxy in Table 2 and Table 3, were used in the comparison study. Although an un-convergent solution was reached, the differences of the resulting *K*_11_ values between the use of *t* = 0.99 and *t* = 0.999 were less than 5%. Furthermore, the analytical *K*_11_ derived in this work is essentially the same as that based on an FEM solution [35], although a finite RVE geometry with different boundary conditions has been used in the FEM approach. More details regarding the FEM approach can refer to [35].

#### 6.3.2. Strength Predictions

**Figure 5:**
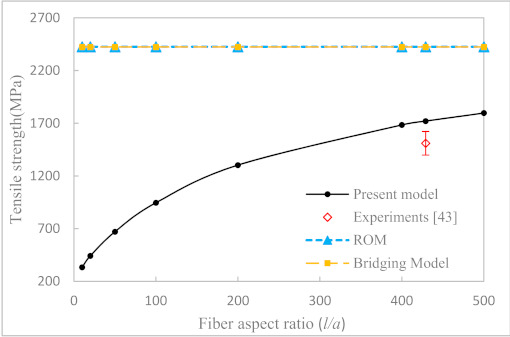
Longitudinal tensile strength of directional T300/Epoxy (*V_f_* = 0.55) SFRC versus fiber aspect ratios.

**Figure 6:**
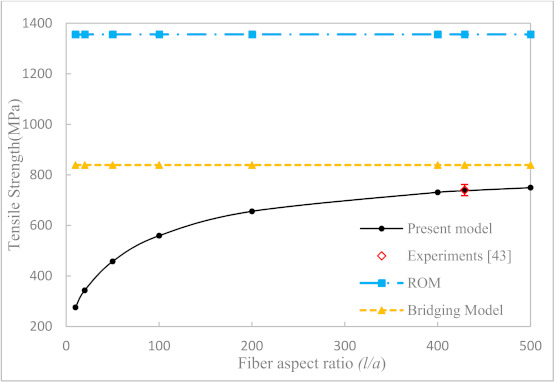
Longitudinal tensile strength of directional E-glass-a/Epoxy (*V_f_* = 0.55) SFRC versus fiber aspect ratios.

**Figure 7:**
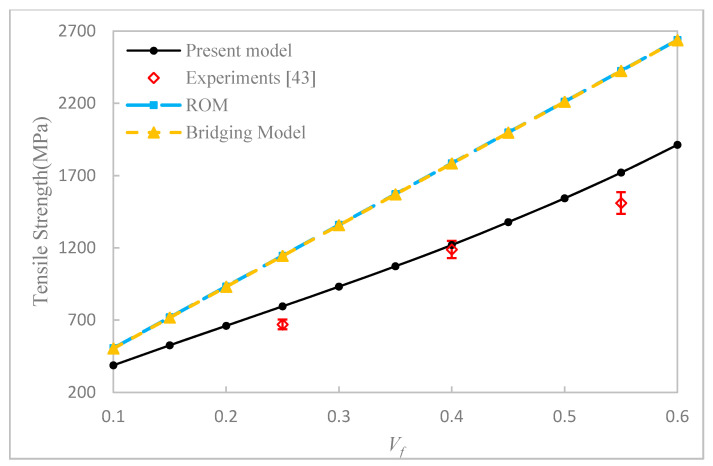
Longitudinal tensile strength of directional T300/Epoxy (*l*/*a* = 429) SFRC versus fiber volume fractions.

Our first example is to study the effect of fiber aspect ratio and fiber volume fraction on the longitudinal tensile strength of uniaxially aligned SFRCs. According to the properties of fiber and matrix given in Table 3, the axial tensile strengths of the UA SFRCs are calculated and are shown in Figure 5, Figure 6 and Figure 7, respectively. The results calculated from ROM and the bridging model for the longitudinal tensile strength of the corresponding CFRCs are also shown in the figures. It can be seen from Figure 5 and Figure 6 that the fiber aspect ratio has an obvious effect on the axial tensile strength of SFRCs. At a fixed fiber volume fraction, the longitudinal tensile strength of SFRCs increases significantly with an increase in the fiber aspect ratio when this ratio is small. The growth rate, however, slows down and reaches a plateau at a larger fiber aspect ratio. From Figure 7, it is found that the longitudinal tensile strength increases nearly linearly with an increase in the fiber volume fraction. Moreover, for T300/Epoxy composite, the predicted results for the strength of the CFRC by the bridging model and ROM are comparable, whereas for the E-glass-a/Epoxy composite with continuous fiber reinforcement, the prediction by Bridging Model seemed more reasonable.

For comparison, the available experimental data taken from Longana et al. [42,43] are shown in the respective figures. In [43], a high-performance–discontinuous fiber (HiPerDiF) method was developed to prepare uniaxially aligned short fiber composites, and the resulting short fibers were nearly directionally arranged in the composite. The tensile strength of the composites was measured and were compared with the prediction results in this paper. All of the correlations between the predictions and the experiments were favorable. The maximum error between the predicted results of this model and the experimental results was 18.6%, and the average error was 11.7%. However, due to a difficulty in chopping a carbon or glass fiber into even smaller segments, the available experiments were all performed with a fiber aspect ratio of 429 [43]. More comparison with experiments of uniaxially aligned short fiber composites having a much smaller fiber aspect ratio, i.e., less than 100, can be expected in the future.

A randomly oriented E-glass-b/PA6.6 SFRC investigated in Ha et al. [27] was chosen as the second illustration example in this work. The fiber aspect ratio was 25. The constituent elastic properties and other input data required are summarized in Table 2. Using these, the prediction for the tensile strength of the composite was carried out as per the procedure described in Section 5.3. Results are summarized in Table 4.

It can be seen that the prediction with all of the SCFs taken into account was the best correlated with the experiments, whereas the predicted strengths without any SCF used to modify the homogenized stresses were in poorest agreement with the experimental results. The use of only a longitudinal tensile SCF of the matrix can significantly improve the prediction accuracy. It is noted that the incorporation of the other directional matrix’s SCFs only resulted in a small improvement in the prediction accuracy. Therefore, the longitudinal tensile SCF of the matrix in an SFRC plays a dominant role in the prediction of its strength subjected to a tensile load. Although obvious improvement has been achieved by using the true stress concept of this work, a noticeable error still exists in Table 4 with the low fiber content. One possible reason may be attributed to the ignorance of matrix plasticity. In this work, both the fiber and matrix are assumed to be linearly elastic until rupture. The influence of matrix plasticity on the load sustaining ability of the composite with a low fiber content is bigger than that with a high fiber content. Another reason may be due to the assumption of the perfect interface bonding assumption. Better correlation can be expected if both issues are taken into account.

**Figure 8:**
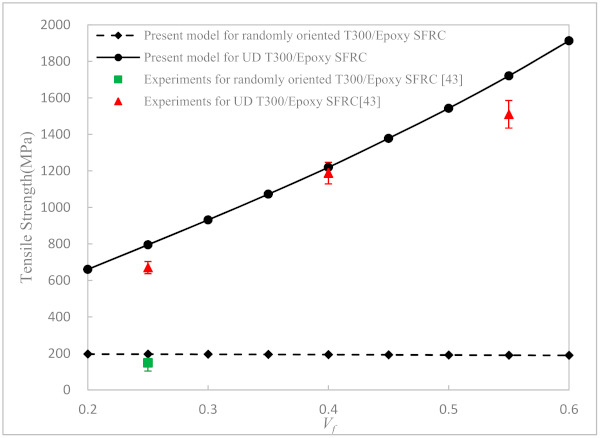
Axial tensile strength of randomly oriented and UD T300/Epoxy (*l*/*a* = 429) SFRC versus fiber volume fraction.

In order to study the tensile strength varied with the fiber orientation, the randomly and uniaxially oriented T300/Epoxy SFRCs at different volume fractions are considered. The predicted strengths are plotted in Figure 8. The measured data taken from [43] are also shown in the figure. It is found that the influence of the fiber orientation is significant. At a certain volume fraction, the tensile strength of the randomly oriented SFRC decreased greatly compared to that of the uniaxial SFRC. In addition, the fiber volume fraction has a bigger effect on the ultimate strength of the uniaxially aligned SFRC than on that of the randomly aligned SFRC. With the fiber volume fraction increased, the strength of the uniaxially aligned SFRC increased significantly, while that of the randomly oriented SFRC was almost unchanged. The predicted strengths of our theory are in reasonably good agreement with the experimental results of both the SFRCs.

## 7. Conclusions

Essentially, every failure of an SFRC results from a matrix failure, and thus the conversion from the homogenized stresses of the matrix into true values is fundamental. In this paper, the conversion for the matrix longitudinal stress component was achieved. The analytical formulae for the longitudinal tensile SCF of the matrix in an SFRC were derived precisely, and the other directional SCFs were the same as those in a CFRC. Once the true stresses were obtained, a matrix tensile failure in the SFRC with any fiber aspect ratio, any fiber length ratio, and any fiber volume fraction could be detected as though there were no fiber or particle reinforcements. Additionally, only the geometrical and mechanical properties of the fiber and matrix were required. Results showed that the volume fraction, the aspect ratio, and the orientation of the fiber all have significant effects on the tensile strength of an SFRC. The predicted tensile strengths of both the uniaxially and the randomly aligned short fiber composites agreed favorably with the available experimental data.

## Figures and Tables

**Table 1:** Fiber and matrix properties.

Material	*E*_11_ (GPa)	*E*_22_ (GPa)	*ν*_12_	*G*_12_ (GPa)	*G*_23_ (GPa)	*σ_u,t_* (MPa)	*σ_u,c_* (MPa)
E-Glass fiber [37]	80	80	0.2	33.33	33.33	2150	1450
LY556 matrix [37]	3.35	3.35	0.35	1.24	1.24	80	120

**Table 2:** Fiber and matrix properties.

Material	*E*_11_ (GPa)	*E*_22_ (GPa)	*ν*_12_	*G*_12_ (GPa)	*G*_23_ (GPa)	*σ_u,t_* (MPa)	*σ_u,c_* (MPa)
T300 fiber [41]	225	15	0.2	15	7	4344	-
E-glass-a fiber [41]	73	73	0.2	30.42	30.42	2400	-
E-glass-b fiber [27]	72	72	0.22	29.51	29.51	1500	-
Epoxy matrix [41]	4	4	0.35	1.48	1.48	80	150
PA6.6 matrix [27]	3.3	3.3	0.35	1.22	1.22	80	145

**Table 3:** Geometric parameters and SCFs of 3 composite systems.

Material System	*a* (μm)	*ξ*	*h/a*	*V_f_*	*γ*	$K_{11}^{}$	$K_{22}^{t}$	$K_{12}^{}$	$K_{23}$
T300/Epoxy [41]	3.5	429	5.19 2.60 1.26	0.25 0.40 0.55	1.0248 1.0181 1.0126	1.18 1.08 0.96	1.60 1.85 2.07	0.91 0.96 1.03	1.48 1.65 1.81
E-glass-a /Epoxy [41]	3.5	429	1.35	0.55	1.0126	0.99	3.04	1.02	2.39
E-glass-b /PA6.6 [27]	5	25	10.93 5.16	0.074 0.194	1.4461 1.3005	1.61 1.52	1.43 1.9	0.88 0.88	1.39 1.67

**Table 4:** Comparison of predicted and experimental values on the tensile strength (MPa) of randomly oriented E-glass-b/PA 6.6 SFRC with an aspect ratio *l/a* = 25.

*V_f_*	Predicted (All SCFs Incorporated)	Error (%)	Predicted (No SCF Considered)	Error (%)	Predicted (Only *K*_11_ Considered)	Error (%)	Measured
0.074	148.9	25.7%	184.8	56.1%	153	29.3%	118.4
0.194	162.5	6.7%	213.9	22.9%	189.2	8.7%	174.1

**Figure A1:**
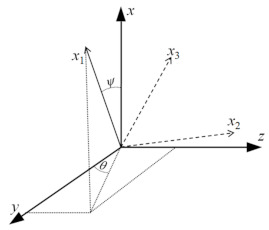
Euler angles to define the orientation of a fiber with respect to global coordinates.

## Data Availability

The data presented in this study are openly available in [Aligned Short Fibre Composites with Nonlinear Behavior] and [Quasi-Isotropic and Pseudo-Ductile Highly Aligned Discontinuous Fibre Composites Manufactured with the HiPerDiF (High Performance Discontinuous Fibre) Technology], reference number [42,43].

## Appendix A. Equations for Coefficients in *a*_11_

(A1) $$\left[\begin{array}{cccc} 0 & 0 & \frac{l}{L}\left[TE_{11}^{f}+\left(1-T\right)E_{m}\right] & \left(1-\frac{l}{L}\right)E^{m}\\ \frac{2\nu^{m}E^{m}}{\left(1+\nu^{m}\right)\left(1-2\nu^{m}\right)} & \frac{2T\nu^{m}E_{m}}{\left(1+\nu^{m}\right)\left(1-2\nu^{m}\right)} & 0 & \frac{E^{m}\left(1-\nu^{m}\right)}{\left(1+\nu^{m}\right)\left(1-2\nu^{m}\right)}\\ \frac{2E^{m}\left[T\nu_{12}^{f}+\nu^{m}\left(1-T\right)\right]}{\left(1+\nu^{m}\right)\left(1-2\nu^{m}\right)} & \frac{-2T\nu_{12}^{f}E^{m}}{1+\nu^{m}} & \frac{2T\nu_{12}^{f}\nu^{m}E^{m}+\left(1-\nu^{m}\right)\left(1-T\right)E^{m}}{\left(1+\nu^{m}\right)\left(1-2\nu^{m}\right)}+TE_{11}^{f} & 0\\ k_{1} & k_{2} & k_{3} & 0 \end{array}\right]\left[\begin{array}{c} C_{1}\\ \frac{C_{2}}{a^{2}}\\ \varepsilon_{L}^{1}\\ \varepsilon_{L}^{2} \end{array}\right]=\left[\begin{array}{c} \sigma_{11}\\ \sigma_{11}\\ \sigma_{11}\\ 0 \end{array}\right]$$

(A2) $$k_{1}=\frac{E_{11}^{f}\left[\begin{array}{l} E^{m}\nu_{12}^{f}\left(1+\nu_{23}^{f}\right)+\nu_{12}^{f}E_{22}^{f}\\ -\left(1+2\nu^{m}\right)\nu^{m}\nu_{12}^{f}E_{22}^{f} \end{array}\right]}{\left[E_{11}^{f}-E_{22}^{f}{\left(\nu_{12}^{f}\right)}_{}^{2}\right]\left(1+\nu^{m}\right)\left(1-2\nu^{m}\right)}-\frac{2\upsilon_{12}^{f}E^{m}}{\left(1+\nu^{m}\right)\left(1-2\nu^{m}\right)}$$

(A3) $$k_{2}=\frac{E_{11}^{f}\left[\nu_{12}^{f}E_{22}^{f}\left(1+\nu^{m}\right)-E^{m}\nu_{12}^{f}\left(1+\nu_{23}^{f}\right)\right]}{\left[E_{11}^{f}-E_{22}^{f}{\left(\nu_{12}^{f}\right)}_{}^{2}\right]\left(1+\nu^{m}\right)}+\frac{2\nu_{12}^{f}E^{m}}{\left(1+\nu^{m}\right)}$$

(A4) $$k_{3}=\frac{E_{11}^{f}\left[E_{22}^{f}{\left(\nu_{12}^{f}\right)}_{}^{2}\left(1+\nu^{m}\right)\left(1-2\nu^{m}\right)+E^{m}\nu^{m}\nu_{12}^{f}\left(1+\nu_{23}^{f}\right)\right]}{\left[E_{11}^{f}-E_{22}^{f}{\left(\nu_{12}^{f}\right)}_{}^{2}\right]\left(1+\nu^{m}\right)\left(1-2\nu^{m}\right)}-\frac{2\nu_{12}^{f}\nu^{m}E^{m}}{\left(1+\nu^{m}\right)\left(1-2\nu^{m}\right)}$$

## Appendix B. Coordinate Transformation Tensor

(A5) $${\left[T_{ij}\right]}_{s}={\left[\begin{array}{cccccc} l_{1}^{2} & l_{2}^{2} & l_{3}^{2} & l_{2}l_{3} & l_{1}l_{3} & l_{1}l_{2}\\ m_{1}^{2} & m_{2}^{2} & m_{3}^{2} & m_{2}m_{3} & m_{1}m_{3} & m_{1}m_{2}\\ n_{1}^{2} & n_{1}^{2} & n_{1}^{2} & n_{2}l_{3} & n_{2}n_{3} & n_{1}n_{2}\\ 2m_{1}n_{1} & 2m_{2}n_{2} & 2m_{3}n_{3} & m_{2}n_{3}+m_{3}n_{2} & m_{1}n_{3}+m_{3}n_{1} & m_{1}n_{2}+m_{2}n_{1}\\ 2n_{1}l_{1} & 2n_{2}l_{2} & 2n_{3}l_{3} & n_{3}l_{2}+n_{2}l_{3} & n_{3}l_{1}+n_{1}l_{3} & n_{2}l_{1}+n_{1}l_{2}\\ 2l_{1}m_{1} & 2l_{2}m_{2} & 2l_{3}m_{3} & l_{2}m_{3}+l_{3}m_{2} & l_{1}m_{3}+l_{3}m_{1} & l_{1}m_{2}+l_{2}m_{1} \end{array}\right]}_{s}$$

(A6) $${\left[T_{ij}\right]}_{c}={\left[\begin{array}{cccccc} l_{1}^{2} & l_{2}^{2} & l_{3}^{2} & 2l_{2}l_{3} & 2l_{3}l_{1} & 2l_{1}l_{2}\\ m_{1}^{2} & m_{2}^{2} & m_{3}^{2} & 2m_{2}m_{3} & 2m_{3}m_{1} & 2m_{1}m_{2}\\ n_{1}^{2} & n_{2}^{2} & n_{3}^{2} & 2n_{2}n_{3} & 2n_{3}n_{1} & 2n_{1}n_{2}\\ m_{1}n_{1} & m_{2}n_{2} & m_{3}n_{3} & m_{2}n_{3}+m_{3}n_{2} & n_{3}m_{1}+n_{1}m_{3} & m_{1}n_{2}+m_{2}n_{1}\\ n_{1}l_{1} & n_{2}l_{2} & n_{3}l_{3} & l_{2}n_{3}+l_{3}n_{2} & n_{3}l_{1}+n_{1}l_{3} & l_{1}n_{2}+l_{2}n_{1}\\ l_{1}m_{1} & l_{2}m_{2} & l_{3}m_{3} & l_{2}m_{3}+l_{3}m_{2} & l_{1}m_{3}+l_{3}m_{1} & l_{1}m_{2}+l_{2}m_{1} \end{array}\right]}_{c}$$

(A7) $$l_{i}=\cos\langle x_{i},x\rangle ,\;m_{i}=\cos\langle x_{i},y\rangle ,\;n_{i}=\cos\langle x_{i},z\rangle ,\;0\leq \psi \leq \pi ,\;0\leq \theta \leq 2\pi$$

(A8) $$\begin{array}{lll} l_{1}=\cos\psi & m_{1}=\sin\psi \cos\theta & n_{1}=\sin\psi \sin\theta \\ l_{2}=\sin\psi & m_{2}=-\cos\psi \cos\theta & n_{2}=-\cos\psi \sin\theta \\ l_{3}=0 & m_{3}=\sin\theta & n_{3}=-\cos\theta \end{array},0\leq \psi \leq \pi ,\;0\leq \theta \leq 2\pi$$

where, e.g., $\langle x_{i}^{k},x\rangle$ denotes the angle between the *k*th fiber local $x_{i}^{k}$, and global *x* coordinates, *ψ* and *θ*, are the Euler angles between the global (*x*, *y*, *z*) and local ($x_{1}^{k},x_{2}^{k},x_{3}^{k}$) coordinates, as shown in Figure A1.
